# Optimizing Multiparametric MRI Protocols for Prostate Cancer Detection: A Comprehensive Assessment Aligned with PI‐RADS Guidelines

**DOI:** 10.1002/hsr2.70172

**Published:** 2024-11-19

**Authors:** Mohammad Hossein Jamshidi, Ali Fatemi, Aida Karami, Sepehr Ghanavati, Durjoy D. Dhruba, Mohammad H. Negarestanian

**Affiliations:** ^1^ Department of Medical Imaging and Radiation Sciences, School of Allied Medical Sciences Ahvaz Jundishapur University of Medical Sciences Ahvaz Iran; ^2^ Department of Physics Jackson State University Jackson Mississippi USA; ^3^ Department of Radiation Oncology Gamma Knife Center Jackson Mississippi USA; ^4^ Department of Medicine, School of Medicine Ahvaz Jundishapur University of Medical Sciences Ahvaz Iran; ^5^ Department of Electrical and Computer Engineering University of Iowa Iowa City Iowa USA; ^6^ Department of MRI, Abu Ali Sina Hospital Shiraz University of Medical Sciences Shiraz Iran

**Keywords:** multiparametric MRI, PI‐RADS, prostate cancer

## Abstract

**Background and Aim:**

Multiparametric magnetic resonance imaging (mpMRI) is recognized as the most indicative method for diagnosing prostate cancer. The purpose of this narrative review is to provide a comprehensive evaluation aligned with the Prostate Imaging and Reporting Data System (PI‐RADS) guidelines, offering an in‐depth insight into the various MRI sequences used in a standard mpMRI protocol. Additionally, it outlines the critical technical requirements necessary to perform a standard mpMRI examination of the prostate, as defined by the PI‐RADS specifications.

**Methods:**

European Society of Urogenital Radiology has released PI‐RADS guideline detailing its suggestions aimed at improving the standards of the procedure. The purpose of this guideline is to establish a standard strategy for MRI protocols and image interpretation, aiming to prevent variability in each of the imaging and interpretation stages.

**Results:**

A standard mpMRI protocol comprises morphological sequences and functional sequences. Morphological sequences which encompass T1‐ and T2‐weighted images, and various functional sequences include diffusion‐weighted imaging, and dynamic contrast‐enhanced MRI. The PI‐RADS recommendations assert that having a standard and uniform protocol for all MRI centers is imperative. Furthermore, the existence of a standardized checklist for interpreting MRI images can foster greater consensus in the process of diagnosing and treating patients.

**Conclusion:**

Standardized protocols and checklists for mpMRI interpretation are essential for achieving greater consensus among radiologists, ultimately leading to improved diagnostic outcomes in prostate cancer.

## 1 Introduction

Currently, prostate cancer holds the distinction of being the most prevalent form of solid cancer in men [1]. Screening and imaging efforts focus on identifying early‐stage diseases with a high biological aggressiveness. The utilization of magnetic resonance imaging (MRI) is currently indispensable in the assessment of the prostate gland [2, 3]. Enhancing conventional MRI with functional MRI, allows for the evaluation of the prostate through multiparametric MRI (mpMRI), establishing itself as the standard imaging method for assessing this organ. This imaging technique plays a vital role in the diagnosis, local staging, and invasive evaluation of prostate cancer, and it is usually the first choice for doctors in the imaging process [4, 5, 6]. The European Society of Urogenital Radiology (ESUR) introduced a guideline known as the Prostate Imaging and Reporting Data System (PI‐RADS) to implement a uniform approach for conducting and documenting prostate MRI examinations [7]. This article provides in‐depth insights into the various MRI sequences utilized in a standard mpMRI protocol. Additionally, it outlines the crucial technical requirements necessary for conducting a standard prostate mpMRI examination, aligning with the specifications defined in PI‐RADS.

## 2 Preprocedure Considerations

Prostate mpMRI typically does not require special preparation and follows a procedure similar to other MRI examinations. However, having clean bowels is recommended. Additionally, it is crucial to ascertain whether the patient has undergone a prostate biopsy and the timing of that procedure. This is particularly important because if the patient has recently had a biopsy and is experiencing bleeding, the presence of blood can pose challenges to the accuracy and interpretation of the results. As a result, PI‐RADS suggests waiting for a minimum of 6 weeks after a biopsy before conducting an MRI for cancer staging purposes [3]. However, there is no necessity to postpone mpMRI following a prior biopsy. Indeed, the primary rationale lies in the fact that the combination of sequences used in mpMRI demonstrates a notable capability to visualize cancerous lesions, even when there is the presence of bleeding [8, 9]. Actually, to prevent any possible bleeding, it is recommended to wait about 6 weeks after the biopsy. However, if necessary and according to the physician's recommendation, this waiting period can be skipped, and the MRI can be performed sooner because the timing of diagnosis is very important. Even in cases of bleeding, the diagnostic power of mpMRI remains very high and can produce images with proper diagnostic accuracy.

## 3 Technical Specifications

### 3.1 MRI Scanners

In prostate mpMRI, scanners with either 3 or 1.5 Tesla magnet strength are utilized. However, choosing a 3T scanner can result in a higher signal‐to‐noise (SNR) ratio compared to a 1.5 Tesla scanner, ultimately enhancing the resolution in imaging. Although contingent upon the pulse sequence and specific implementation details, there could be potential drawbacks at 3 Tesla, including increased power deposition, susceptibility‐related artifacts, and signal heterogeneity [10, 11, 12]. Tackling these issues might lead to a minor uptick in imaging duration and/or a decrease in SNR. Nevertheless, modern 3 Tesla MRI scanners have demonstrated the capability to effectively tackle these challenges. The PI‐RADS committee recommend that the advantages provided by 3 Tesla MRI significantly outweigh these potential concerns [11].

A study shows that 3T mpMRI offers better image resolution and clarity for detecting prostate cancer than 1.5T MRI, with its higher magnetic field strength allowing for more accurate tumor detection, especially in challenging areas like the anterior prostate [13]. In another clinical case study, 3 T mpMRI was able to reveal a significant tumor in the prostate's peripheral zone, which was missed by the lower resolution 1.5T MRI, demonstrating the enhanced diagnostic power of 3 T scanners in locating smaller or more subtle lesions [14].

### 3.2 Coils

To achieve a high SNR, it is preferable to utilize a combination of an external phased array coil with an endorectal coil. This combination allows for enhanced resolution, particularly when employing 1.5 Tesla scanners. While endorectal coils contribute to a better SNR ratio, they may lead to dissatisfaction in some patients. Moreover, for patients who have recently had a biopsy, the use of endorectal coils is not recommended due to the potential risk of bleeding [15, 16]. Hence, it might still be preferable to choose a coil equipped with a high number of receiver channels (16 or more) at 1.5 Tesla, rather than an endorectal coil. For 3 Tesla scanners as well, a single array coil is considered sufficient [17]. Discomfort and pain, along with psychological distress, can be a significant reason for patients' discomfort when using endorectal coils. Therefore, other potential alternatives can be considered. There are a few alternatives to endorectal coils for mpMRI of the prostate [18, 19], including:

- Fully balanced steady‐state free precession (bSSFP): This technique can be used to acquire MRI scans without an endorectal coil, and can offer advantages like better patient tolerance and lower costs.
- Nonendorectal coil MRI is less invasive than endorectal coil MRI, but it may not be an equal substitute. Nonendorectal coil MRI images can be larger due to lower in‐plane resolution.
- Multichannel surface coil imaging
- An 8 channel pelvic phased array

### 3.3 Computer‐Aided Diagnosis (CAD) and Artificial Intelligence

Using CAD technology via specialized software is not mandatory for interpreting mpMRI images. However, it can prove beneficial for post‐processing or image‐filtering purposes. The goal of CAD is to overcome variations between different observers by employing machine learning algorithms that depend on quantitative data analyses [16, 20, 21]. However, incorporating CAD can improve multiple facets of the workflow, such as display, analysis, interpretation, reporting, and communication. Furthermore, CAD can furnish quantitative pharmacodynamics data and improve the ability to detect and differentiate lesions. This is particularly beneficial for radiologists who may have limited experience in interpreting mpMRI images [22]. In recent years, the use of artificial intelligence in the evaluation of medical images has expanded significantly. In the field of mpMRI imaging, machine learning and deep learning algorithms, along with neural networks, have significantly assisted physicians by extracting and categorizing quantitative data [23, 24, 25], including:

- Increasing the MRI images quality
- Improving the diagnosis process
- Enhancing diagnostic accuracy
- Reducing human errors
- Decreasing false negatives
- Shortening the interpretation time

## 4 MpMRI Protocols and Technical Aspects

A standard mpMRI protocol comprises morphological sequences and functional sequences. Morphological sequences which encompass T1‐ and T2‐weighted images, and various functional sequences include diffusion‐weighted imaging (DWI), dynamic contrast‐enhanced (DCE) MRI, and proton spectroscopy. It's important to mention that according to PI‐RADS guidelines, MR Spectroscopy (MRS) is no longer advised as a routine component of prostate mpMRI [26, 27]. The protocol selection is generally influenced by factors such as the physician's preferences and the patient's physical condition, although a standard framework exists. In the following sections, we will delve into the sequences utilized in mpMRI, with a focus on their technical specifications.

### 4.1 T1‐Weighted Sequences

**Figure 1:**
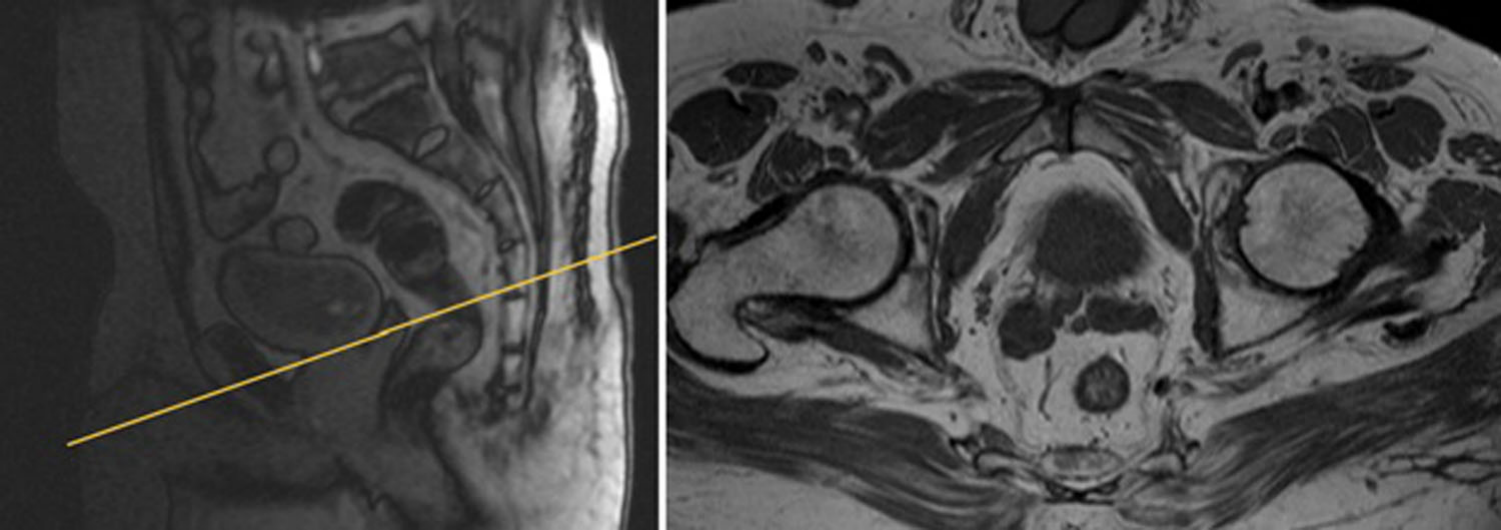
T1‐weighted GE image of the prostate in axial plane.

T1‐weighted imaging is employed to assess regional lymph nodes and bone structures, with its primary purpose being the detection of hemorrhages associated with biopsy, which can potentially obscure cancerous lesions. It is important to note that this sequence is not particularly effective in identifying specific prostate cancer foci, as prostate cancer typically does not produce significant alterations in T1‐weighted imaging [28]. In this sequence, axial or coronal spin‐echo (SE) or gradient‐echo (GE) sequences are used with a wide field of view (FOV) to exclude potential bleeding resulting from biopsy, as illustrated in Figure 1.

### 4.2 T2‐Weighted Sequences

**Figure 2:**
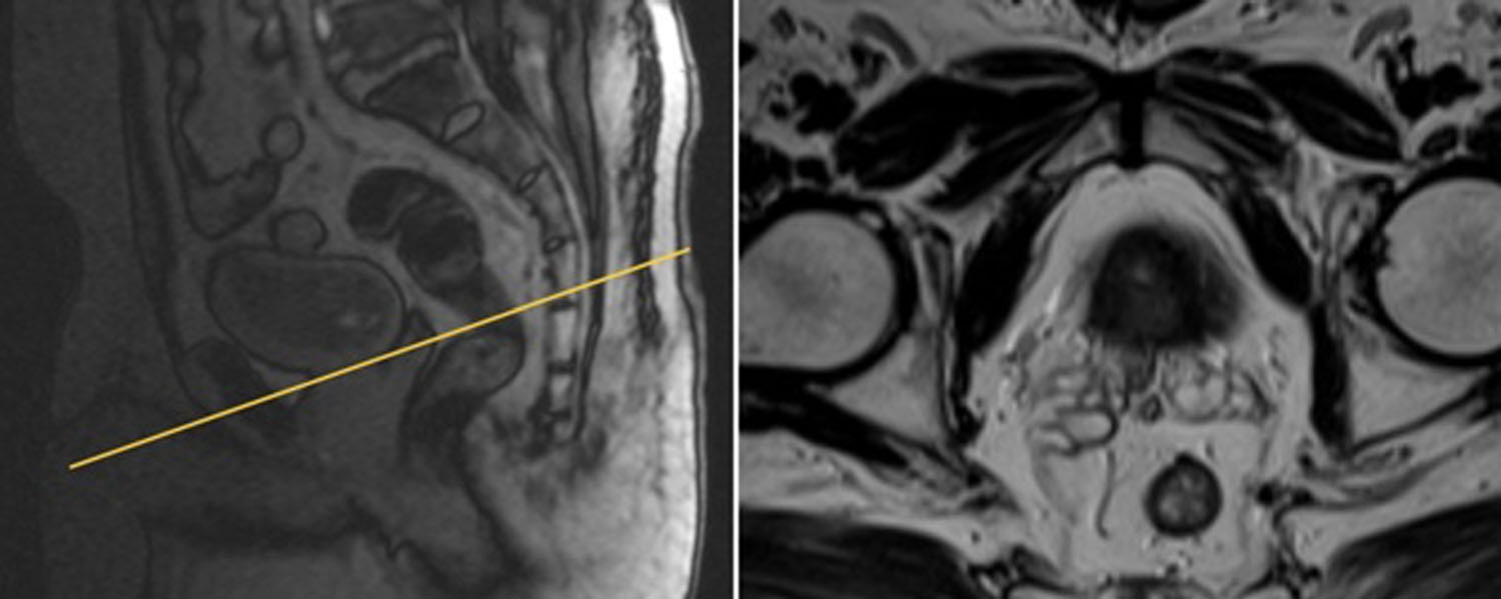
T2‐weighted with small FOV high resolution of prostate axial plane.

The T2‐weighted sequence generates anatomical images that provide excellent soft‐tissue contrast, depicting the prostate gland. T2‐weighted images are typically acquired in the axial, coronal, and sagittal planes with a smaller FOV, as illustrated in Figure 2. This sequence plays a crucial role in delineating the zonal anatomy of the prostate, identifying abnormalities, and assessing for seminal vesicle invasion, extraprostatic extension, and nodal involvement [29, 30]. There are two approaches to T2‐weighted imaging in prostate MRI. The first utilizes axial multiplanar 2D fast spin echo (FSE) sequences with a small FOV ranging from 12 to 20 cm, a slice thickness of 3 mm, and in‐plane dimensions of ≤ 0.7 mm (phase) *x* ≤ 0.4 mm (frequency). It is advisable to include at least one additional plane (coronal or sagittal) [7]. These two sequences (axial + coronal or sagittal) are employed for calculating prostate volume. The second approach uses a single 3D FSE acquisition with isotropic voxels and contiguous thin‐section slices of ≤ 1 mm in the axial plane. Additionally, reconstructions in the coronal and sagittal planes are performed [31].

### 4.3 Diffusion Weighted Imaging (DWI)

**Figure 3:**
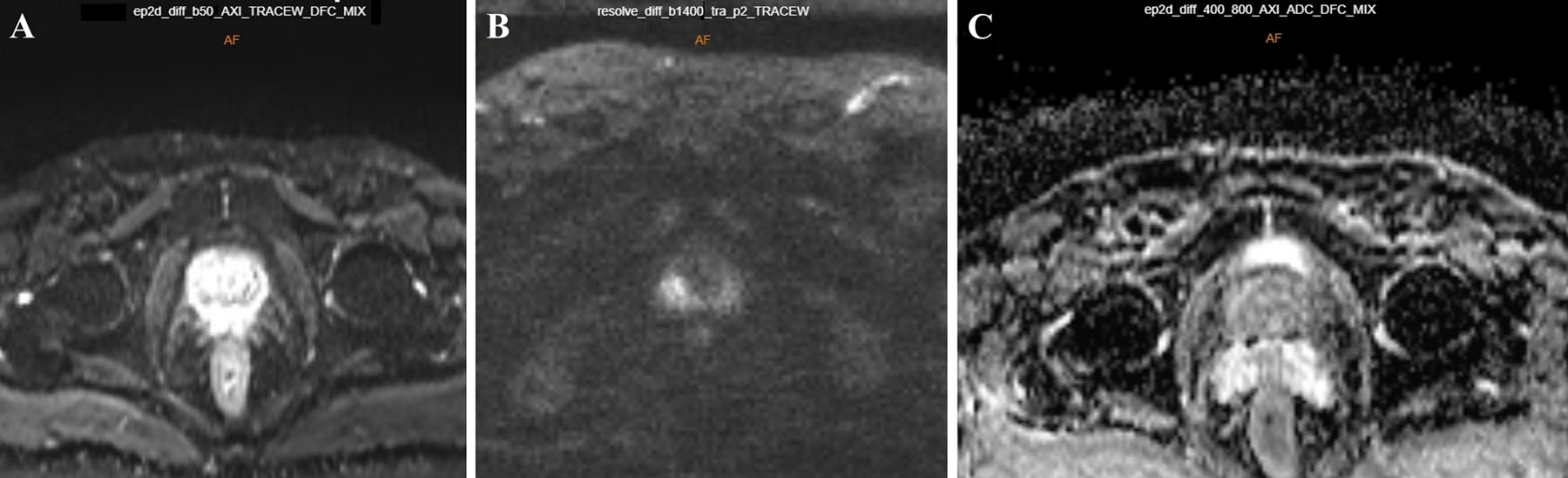
DWI acquired at b‐value 50 s/mm^2^ (A), *b*‐value 1400 s/mm^2^ (B) with ADC map (C). High *b*‐values result in the suppression of the background normal prostate signal, enabling the visualization of cancerous lesions as bright areas.

DWI measures the movement of water molecules in tissue. In a healthy prostate, water molecules move freely, while prostate cancer restricts this motion, leading to a lower apparent diffusion coefficient (ADC) [32]. DWI uses different b‐values (magnetic gradient strengths) to generate an ADC map. Higher *b*‐values, starting at 1400 s/mm² for 1.5T MRI and 2000 s/mm² for 3T MRI, enhance the detection of prostate cancer by reducing background signals from healthy tissue [33, 34, 35]. Cancerous areas appear bright in high *b*‐value images and dark on ADC maps, whereas healthy tissue shows the opposite pattern, as illustrated in Figure 3.

For ease of interpretation, it is recommended to maintain slice thicknesses and locations consistent with T2‐weighted images. Adherence to specific technical parameters is essential, including an echo time (TE) of ≤ 90 ms, a repetition time (TR) of ≥ 3000 ms, a slice thickness of ≤ 4 mm, a FOV ranging from 16 to 22 cm, and in‐plane dimensions of ≤ 2.5 mm for both phase and frequency. According to PI‐RADS guidelines, acquiring at least two *b*‐value images is essential for accurate ADC calculation. These technical guidelines aim to standardize and optimize the imaging process, ensuring the reliability of results in prostate imaging assessments. It is advisable to configure the lower b‐value within the range of 0–100 s/mm² for optimal imaging outcomes, with a preference for values between 50 and 100 s/mm². The optimal selection for the higher *b*‐value lies in the range of 800–1000 s/mm². Additionally, acquiring ultrahigh *b*‐value images, specifically within the range of 1400–2000 s/mm², is recommended. This approach is particularly advantageous for improving the visibility of cancers, especially in prostate cancer. Including these specific *b*‐values in the imaging protocol aims to maximize sensitivity and accuracy in the detection of prostate cancers, with a particular emphasis on those of clinical importance within the transition zone [7, 36].

### 4.4 Dynamic Contrast‐Enhanced (DCE)‐MRI

In prostate imaging, DCE sequences are employed to investigate tumor angiogenesis. This involves the analysis of variations in the rates and levels of contrast agent absorption and elimination between malignant and nonmalignant prostate tissue. By capturing the dynamic changes in contrast enhancement throughout the imaging procedure, DCE‐MRI sequences provide crucial insights into the vascular patterns within the prostate. This information aids in distinguishing cancerous from noncancerous tissues based on their angiogenic characteristics [36]. DCE‐MRI involves rapidly acquiring a series of T1‐weighted images following the intravenous administration of a gadolinium‐based contrast agent. This sequence allows for assessing contrast enhancement's intensity and temporal dynamics [37]. The hallmark of cancer is characterized by a distinguishing feature of early enhancement accompanied by heightened intensity in T1‐weighted images. DCE‐MRI alone has demonstrated sensitivity and specificity for prostate cancer detection, falling within 46%–90% and 74%–96% [38].

DCE‐MRI is typically performed utilizing a T1‐weighted GE sequence and prefers 3D sequences over 2D sequences to achieve comprehensive volumetric coverage In DCE‐MRI, it is essential to ensure craniocaudal coverage that matches that of T2‐weighted and DWI images, even if there is a possibility of a reduction in in‐plane resolution. PI‐RADS guidelines recommend a high temporal resolution, with rapid and repeated scanning of the entire prostate every 7–10 s, with at least 2 min of continuous scanning considered necessary. A temporal resolution of < 15 s is deemed sufficient. Additionally, fat suppression is recommended to enhance lesion visibility [39, 40, 41]. In addition, the quality of the obtained images is crucial. Therefore, studies that assess image quality across different protocols and scanners, using standardized comparisons, can be highly valuable. For instance, in a study examining 71 different scanners, DCE‐MRI sequences demonstrated the lowest concordance with PI‐RADS criteria [42].

## 5 Interpretation and Reporting

**Figure 4:**
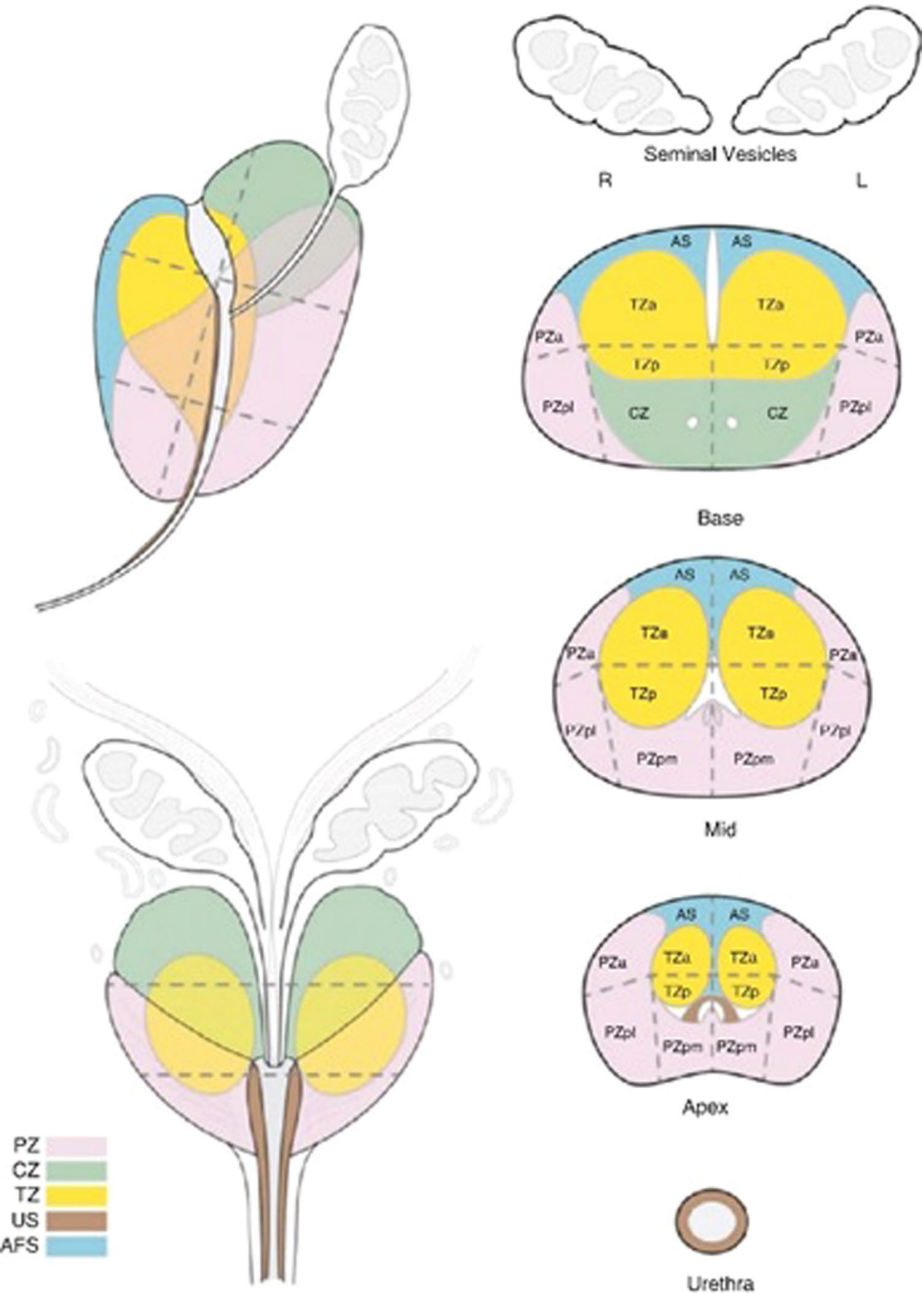
Sector map for topographical location of lesions (PI‐RADS v2 version). Using a sector map for reporting allows easier understanding between clinicians to understand location of lesion and plan intervention and therapy. AFS (AS) anterior fibromuscular stroma, a, anterior; CZ, central zone; l, lateral; m, medial; p, posterior; PZ, peripheral zone; TZ, transition zone; US, urethral sphincter.

According to Table 1, PI‐RADS assessment employs a 5‐point scale based on the likelihood that a combination of mpMRI findings correlates with the presence of clinically significant cancer in each lesion within the prostate gland [7]. The scale aids in categorizing identified lesions, thereby guiding clinical decision‐making regarding the need for further evaluation or intervention. The report must comprise a description of PI‐RADS lesions, along with location‐based scoring as depicted on the 39‐sector map, as illustrated in Figure 4 [7], measurements of the lesion and prostate gland volume, T1‐weighted analysis, assessment of extra‐glandular extension, evaluation of lymph nodes and bones, lesion description, evaluation of pelvic bones. Additionally the final PI‐RADS score, spanning from 1 to 5, signifies the likelihood of clinically relevant prostate cancer, along with the included conclusion and recommendations. Certainly, to enhance the diagnostic process, CAD technology can function as an intelligent assistant, process imaging data, offer quantitative information, and thereby improve the accuracy and efficiency of radiologists [43, 44].

**Table 1:** PI‐RADS assessment categories.

PI‐RADS 1
▪ Very low risk of prostate cancer (prostate cancer is highly unlikely)
PI‐RADS 2
▪ Low risk of prostate cancer (prostate cancer is unlikely)
PI‐RADS 3
▪ Intermediate risk of prostate cancer (the presence of prostate cancer is equivocal)
PI‐RADS 4
▪ High risk of prostate cancer (prostate cancer is likely)
PI‐RADS 5
▪ Very high risk of prostate cancer (prostate cancer is highly likely)

## 6 Accuracy of mpMRI Protocols

Before the implementation of the scoring system by the PI‐RADS committee to evaluate mpMRI, numerous investigations had already been conducted to explore the significance of mpMRI in the identification and categorization of prostate cancer. In these studies, the T2‐weighted sequence was assessed independently, followed by its combination with DWI and DCE‐MRI, and ultimately as a combination of all three sequences. The results of the patient's biopsy were considered the gold standard for an accurate comparison [45, 46]. The results of most of these studies showed that the combination of T2‐weighted with DWI is more effective than other protocols [47, 48, 49, 50]. A meta‐analysis showed that this approach was found to have a specificity of 0.88 and a sensitivity of 0.74 [50]. In summary, the addition of DWI enhances both sensitivity and specificity for detection.

## 7 Limitations

While the mpMRI technique has therapeutic capabilities and widespread use in recent years, it is not without limitations. If mpMRI testing is performed with a field strength < 1.5 Tesla [51], then there could be a technical obstacle to achieving the recommended parameters according to the PIRADS guidelines. In addition, DWI and DCE‐MRI are highly susceptible to motion artifacts from prostate spasms and muscle movements. This might compromise the image quality. Severely obese patients can pose additional limitations due to the sheer thickness of the adipose tissue. This may cause greater coil‐to‐prostate distance resulting in deterioration of the image quality. Excessive rectal gas can hinder the interpretation of diffusion‐weighted sequences, making the scan challenging to analyze. Moreover, the effectiveness of mpMRI studies highly depends on the radiologist's expertise. A complete patient profile is required to properly interpret the morphological data obtained through mpMRI procedures.

## 8 Challenges and Suggestions

In the process of mpMRI for prostate cancer, challenges are categorized into two main groups. The first category relates to the acquisition of images. Typically, when conducting the T2‐weighted sequence, a wrap artifact may occur, and this can be avoided by expanding the FOV. Additionally, minimizing imaging time and increasing the echo train length can be effective in reducing movement artifacts in this sequence. In the DWI sequence, there is a notable risk of susceptibility artifact. To prevent this, it is essential for the patient's intestines, particularly the rectum, to be empty before imaging. If gas is present in the intestine, using an endorectal coil can displace it. The most noteworthy imaging challenge is associated with the DCE‐MRI sequence, where rectal motion artifact is common, and the use of left‐right phase encoding can help alleviate this problem.

The second category pertains to the interpretation of images. Irrespective of a radiologist's expertise, diagnosing prostate cancer can be intricate due to factors like prostate size, cancer type, and grade. Imaging symptoms may vary, posing challenges, particularly for less experienced radiologists. Therefore, having a highly accurate auxiliary tool is crucial. Artificial intelligence and machine learning can function as effective assistants in this domain. By inputting image data from MRI, along with biopsy and pathology reports for various prostate cancer types, an intelligent model can be developed. Trained with MRI images and biopsy results, this intelligent model will be valuable in supporting radiologists during the interpretation and can detect various cancers, including those in their early stages.

## 9 Conclusion

This investigation critically examined and presented a comprehensive evaluation of the recommendations put forth by the ESUR, and further explored a thorough and profound analysis of the diverse MRI sequences employed in a typical mpMRI protocol for the detection and diagnosis of prostate cancer. All ESUR recommendations, including patient preparation, equipment, imaging protocols, and even image interpretation, are thoroughly explained, and the challenges associated with each are discussed. These suggestions firmly declare that mpMRI plays a vital role in the diagnosis and treatment of prostate cancer. While unforeseen circumstances may arise in practice or clinical settings, and sometimes patients may present unique cases, having a standard and uniform protocol for all MRI centers is imperative. Furthermore, the existence of a standardized checklist for interpreting MRI images can foster greater consensus in the process of diagnosing and treating patients.

## Author Contributions

**Mohammad H. Jamshidi:** conceptualization, visualization, supervision, writing–review and editing, writing–original draft, validation, project administration. **Ali Fatemi:** conceptualization, writing–original draft, validation, data curation, writing–review and editing. **Aida Karami:** writing–original draft, data curation, conceptualization. **Sepehr Ghanavati:** data curation, writing–original draft. **Durjoy D. Dhruba:** writing–original draft. **Mohammad H. Negarestanian:** data curation.

## Conflicts of Interests

The authors declare no conflicts of interest.

## Transparency Statement

The lead author Mohammad Hossein Jamshidi affirms that this manuscript is an honest, accurate, and transparent account of the study being reported; that no important aspects of the study have been omitted; and that any discrepancies from the study as planned (and, if relevant, registered) have been explained.

## Data Availability

Data sharing is not applicable to this article as no datasets were generated or analyzed during the current study. All data are available in the main text. All authors have read and approved the final version of the manuscript. Mohammad Hossein Jamshidi had full access to all of the data in this study and takes complete responsibility for the integrity of the data and the accuracy of the data analysis. No data set available as no new data were generated.
